# Advances in Metal Microstructure Simulation and Analysis

**DOI:** 10.3390/ma19102072

**Published:** 2026-05-15

**Authors:** Meng Liu, Hongrui Zhou, Hui Jiang, Caixu Yue

**Affiliations:** 1 School of Mechanical and Power Engineering, Harbin University of Science and Technology, Xue Fu Street, Harbin 150080, China; 2 The College of Mechanical and Electrical Engineering, Qiqihar University, Wen Hua Street, Qiqihar 161000, China; m494949494@163.com; 3 Qiqihar Heavy CNC Equipment Co., Xin Cheng Street, Qiqihar 161005, China

**Keywords:** changes in metal microstructure, discrete field model, continuous field model, microstructure

## Abstract

Numerical simulation of metal microstructure evolution is essential for material design and performance optimization. This paper reviews major simulation methods for key evolution mechanisms, including recrystallization, grain growth, slip, twinning, and phase transformation. The reviewed methods are classified into atomistic models, discrete-field models, and continuous-field models. Molecular dynamics (MD) is discussed as an independent atomistic approach, with emphasis on its role in revealing atomic-scale mechanisms, calibrating mesoscale parameters, and bridging atomistic, mesoscale, and continuum simulations. Discrete-field methods, including Monte Carlo, cellular automata, and vertex models, are compared with continuous-field methods, including artificial neural networks, phase field models, finite element methods, and level-set methods. Furthermore, a semi-quantitative evaluation matrix based on accuracy, computational cost, scalability, and applicability is established to clarify the practical trade-offs among different methods. The results show that no single method is universally optimal; instead, method selection should depend on the dominant physical mechanism, target length scale, required accuracy, and available computational resources. This review provides methodological guidance for multiscale microstructure simulation and supports future applications in precision machining, additive manufacturing, and process parameter optimization.

## 1. Introduction

The dynamic evolution mechanisms of metallic microstructures serve as the critical link connecting material processing techniques with macroscopic properties [1,2]. From recrystallization processes in high-temperature forging to melt pool solidification in additive manufacturing, and from defect evolution under irradiation damage to phase transformation behaviors in service environments, the nonlinear responses of microstructures across spatiotemporal scales fundamentally determine the mechanical properties, durability, and functional characteristics of materials [3,4,5,6]. While conventional experimental methods can capture microstructural features at specific spatiotemporal nodes, they remain inadequate for comprehensively resolving the dynamic mechanisms and critical transition patterns in evolution processes under coupled multi-physical fields [7,8,9]. This cognitive bottleneck has spurred the rapid development of numerical simulation technologies. Through establishing physics-driven computational models, researchers can now reconstruct the evolutionary landscape of microstructures in virtual environments, unveiling the kinetic essence of nucleation, growth, and phase transformation processes.

The evolution of metallic microstructures constitutes a complex process involving multiple interrelated mechanisms, including recrystallization, grain growth, slip, twinning, and phase transformations. Recrystallization [10,11,12] refers to the formation of new strain-free grains through thermal activation in cold-worked metals, typically occurring at specific annealing temperatures. This process not only eliminates internal stresses but also significantly enhances the material’s plasticity and toughness [13,14,15]. Grain growth [16,17,18,19] describes the phenomenon where grains gradually enlarge via boundary migration at elevated temperatures, generally leading to reduced strength yet improved ductility. Slip [20,21,22], as a primary plastic deformation mechanism, operates through dislocation motion within crystal lattices, with its directionality and magnitude governed by both crystallographic orientation and external loading conditions. Twinning [23] represents another deformation mechanism, particularly dominant under low-temperature or high-strain-rate conditions, characterized by mirror-symmetric shear deformation between crystallographic regions. Phase transformations [24,25] involve structural transitions between distinct crystalline states driven by thermal or mechanical stimuli. These mechanisms collectively orchestrate the dynamic reorganization of metallic microstructures, ultimately dictating the material’s macroscopic performance characteristics.

Traditional experimental methods have long dominated the study of metallic microstructures, yet their inherent reliance on posterior observation and analysis makes it difficult to predict microscopic response patterns under complex external fields (such as multi-axial stress and high thermal gradients) prior to experimentation. This “trial-and-error” research paradigm results in significant blindness in experimental design, particularly when confronting extreme operating conditions or novel alloy systems, where exorbitant prototyping costs and protracted cycles severely constrain material development efficiency. Within this context, computational simulation methods have evolved from auxiliary tools to an indispensable research paradigm due to their a priori predictive capability and multi-dimensional analytical characteristics, providing the core driving force for establishing a closed-loop “theoretical prediction–experimental verification” research system.

This paper focuses on the methodological evolution of metallic microstructure simulation technologies, systematically categorizing them into discrete-field and continuous-field systems based on the mathematical essence of physical modeling. Discrete-field models reveal dynamic competitive mechanisms in microstructural evolution by constructing evolution rules for discretized mesoscopic units (e.g., grains, dislocation lines): Monte Carlo (MC) methods simulate grain boundary migration based on energy minimization principles; Cellular Automata (CA) capture recrystallization nucleation and growth processes through local evolution rules; and Vertex models excel in describing topological reconstruction behaviors in polycrystalline systems. Continuous-field models establish governing equations for field variables via continuum medium assumptions: Phase Field (PF) methods employ order parameter gradients to describe phase interface evolution; the Finite Element Method (FEM) correlates macroscopic deformation with microscopic responses through mechanical constitutive relationships; and Artificial Neural Networks (ANNs) leverage data-driven patterns to uncover complex nonlinear correlations.

**Figure 1:**
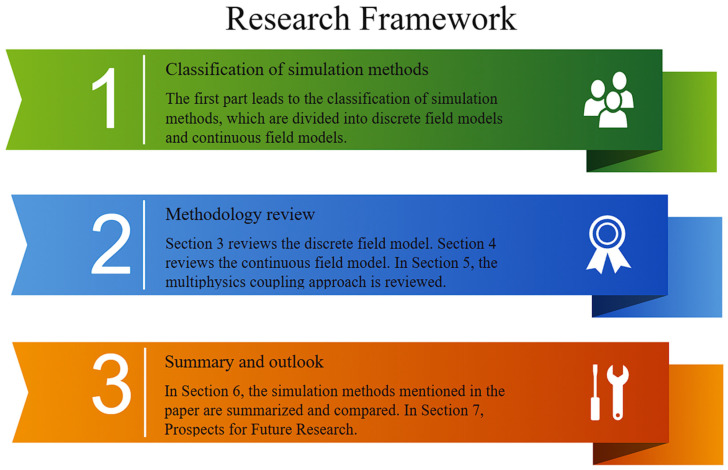
This paper examines the framework diagram.

This paper systematically reviews metallic microstructure simulation methods and proposes an innovative classification framework, adopting a tripartite structure of “theoretical construction–methodological analysis–forward integration”: Part I (Section 1 and Section 2) establishes theoretical foundations for microstructural evolution, introducing a discrete-field/continuous-field dichotomy based on mathematical-physical modeling essence. Part II (Section 3, Section 4 and Section 5) conducts in-depth methodological analysis, with Section 3 exploring discrete-field models (Monte Carlo, Cellular Automata), Section 4 dissecting continuous-field models (Phase Field, Finite Element), and Section 5 focusing on cross-scale breakthroughs in multi-physics coupling algorithms. Part III (Section 6 and Section 7) constructs method evaluation matrices and outlines future directions, where Section 6 further establishes a semi-quantitative comparison framework based on four criteria, namely prediction accuracy, computational cost, scalability, and applicability. By integrating reported validation errors, correlation coefficients, computational complexity, dimensional extensibility, and typical application scenarios, the practical trade-offs among MC, CA, Vertex, ANN, PF, FEM, Level Set, and coupled models are clarified. Section 7 proposes technological evolution paths addressing cutting-edge fields like high-throughput computing and quantum-classical hybrid paradigms. This structural design achieves a transition from methodological deconstruction to disciplinary paradigm reconstruction, providing systematic research frameworks for next-generation computational materials science. Figure 1 shows the research framework of this paper.

## 2. Classification of Numerical Simulation Methods

**Figure 2:**
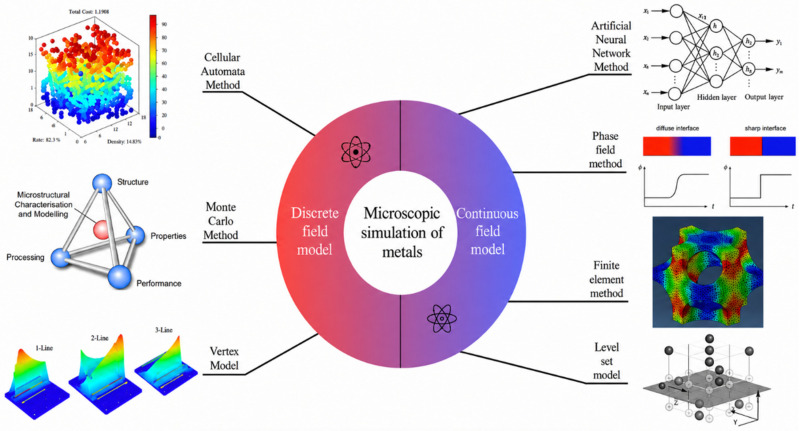
Numerical simulation methods for microscopic simulation of metals.

In the study of metallic microstructure simulation, experimental methods face significant limitations when observing microstructural evolution, particularly under complex deformation conditions, as they struggle to provide comprehensive and continuous microscopic information. Consequently, numerical simulation has emerged as a critical approach for investigating metallic microstructural evolution. The primary numerical simulation methods in this field include Artificial Neural Networks (ANNs), Monte Carlo (MC), Cellular Automata (CA), Vertex models, Phase Field methods, the Finite Element Method, and the Level Set Method. From a mathematical modeling perspective, these approaches can be categorized into two distinct classes, as illustrated in Figure 2. Although this review mainly classifies microstructure simulation methods into discrete-field and continuous-field models, atomistic simulation methods are also indispensable for understanding microstructure evolution. Molecular dynamics (MD) is not included in the above two categories because it directly resolves atomic motion rather than describing microstructure evolution through field variables or mesoscopic discrete rules. Therefore, MD is discussed separately as a complementary atomistic method, focusing on its role in mechanism discovery, parameter calibration, and multiscale coupling.

(1) Discrete-Field Models

Discrete-field models are mathematical frameworks that discretize continuous spatial and temporal domains to simulate systems with inherent discrete characteristics. The Monte Carlo method employs stochastic sampling and statistical analysis to estimate system behaviors through random trials, particularly effective in probabilistic simulations. Cellular Automata operate through discrete spatial grids and localized transition rules, where cells with finite states evolve synchronously based on neighborhood interactions, enabling pattern emergence studies. Vertex models specialize in biological tissue simulation by representing cellular structures through interconnected vertices that dynamically reconfigure via mechanical interactions. These approaches share fundamental discretization principles, transforming complex continuum phenomena into computationally tractable discrete-state systems while preserving essential physical/biological mechanisms.

(2) Continuous-Field Models

Continuous-field models mathematically describe spatially continuous phenomena through field variables defined over continuous domains. Artificial Neural Networks (ANNs) achieve continuous input–output mapping via multilayer architectures and nonlinear activation functions, enabling approximation of complex continuous functions for field prediction. The Phase Field method introduces diffusive phase field variables to implicitly track material interfaces, effectively simulating phase transitions (liquid–solid/solid-state transformations) without explicit interface tracking. The Finite Element Method (FEM) discretizes physical domains into geometrically adaptable elements, solving partial differential equations through piecewise polynomial approximations to handle complex geometries and material nonlinearities. The Level Set Method represents evolving interfaces as zero-level contours of higher-dimensional functions, excelling in capturing topological changes like fracture propagation. These approaches collectively address continuum-scale challenges through mathematical continuity principles while employing distinct strategies: ANNs leverage data-driven universal approximation, Phase Field utilizes thermodynamic variational formalisms, the FEM applies spatial discretization, and Level Set employs implicit interface parameterization, all converging to resolve multi-physics coupling in continuous field simulations.

## 3. Discrete Field Model

### 3.1. Monte Carlo Method

The Monte Carlo method (MC) is a statistical numerical simulation technique rooted in discrete probabilistic sampling principles, with its mathematical foundation established in statistical estimation theory and the Central Limit Theorem. This method’s core principle lies in constructing stochastic process models with defined probabilistic characteristics, deriving approximate analytical solutions for complex systems through large-scale random sampling experiments to obtain statistical distribution features of parameters. As a non-deterministic numerical algorithm, MC effectively addresses challenges intractable to conventional analytical methods—such as high-dimensional integration, stochastic process simulation, and nonlinear system analysis—by leveraging the convergence properties of probability density functions [26,27,28,29,30].

Historically developed by Stanislaw Ulam and John von Neumann at Los Alamos National Laboratory in the 1940s, MC was initially applied to probabilistic modeling of neutron transport in nuclear reactors. A breakthrough emerged in the 1950s with N. Metropolis’s team proposing the Metropolis sampling criterion, which advanced importance sampling techniques through Markov Chain Monte Carlo (MCMC) methods, dramatically enhancing sampling efficiency for complex systems and establishing MC’s foundational role in microscopic material simulations. In statistical physics, MC has been successfully deployed for thermodynamic property calculations of many-body systems, critical phase transition predictions, and solid–liquid interfacial free energy analyses, thereby constructing a systematic numerical framework for modern computational materials science. Table 1 shows the application history of the MC in microscopic simulation [31,32,33,34,35].

In Monte Carlo simulations of metallic microstructure evolution, the lattice-based Potts model [36] is typically employed, with core implementation steps outlined as:

(1) Construct a 3D discretized grid representing the polycrystalline system, where each lattice site is assigned an integer variable denoting crystallographic orientation;
(2) Define the system Hamiltonian;
(3) Randomly select lattice sites for virtual orientation flips and compute energy variation ΔE;
(4) Update states probabilistically according to the Metropolis criterion;
(5) Apply periodic boundary conditions to eliminate size effects, iterating system evolution using Monte Carlo Steps (MCS) as dimensionless time units. This approach statistically samples microstate transition pathways to quantitatively reveal complex kinetic behaviors like grain boundary migration, recrystallization, and abnormal grain growth. Its validity has been experimentally confirmed in grain coarsening studies of austenitic stainless steels and nickel-based superalloys [37].

The fundamental formula governing metallic microstructure evolution in Monte Carlo simulations derives from the Potts model Hamiltonian [38]:

$$H=-J\sum_{\left\langle i,\;j\right\rangle }\;\delta (q_{i},q_{j})+\sum_{k}\;E_{k}(q_{k})$$

Here, *J* is the grain boundary energy coefficient. *δ*(*q_i_*, *q_j_*) is the Kronecker function. It equals 1 when the crystallographic orientation parameters *q_i_* and *q_j_* of adjacent lattice points *i* and *j* are the same, and 0 if they are different. ∑〈*i*, *j*〉 means we sum over all pairs of nearest-neighbor lattice points [39]. The second term *E_k_*(*q_k_*) can account for external field energy, like strain energy or chemical potential gradient [40]. We use the Metropolis criterion to decide the probability of state update.

$$P(\Delta E)=\{\begin{array}{cc} 1 & \Delta E\leq 0\\ exp(-\frac{\Delta E}{KT}) & \Delta E>0 \end{array}$$

where *K* is the Boltzmann constant and *T* the simulation temperature. Spatially, Monte Carlo simulations topologically resolve the final distribution and size of recrystallized grains. Temporally, they additionally enable quantitative analysis of temporal evolution in grain size, boundary mobility, and other microstructural characteristics.

Peczak [41] first achieved quantitative prediction of dynamic recrystallization (DRX) flow curves using a spin-lattice-based Monte Carlo model. The study revealed that when using the Zener–Hollomon parameter as a scaling factor, simulated stress–strain curves exhibited a transition from multimodal to unimodal states consistent with experimental observations, elucidating the regulatory mechanism of thermal activation processes on DRX critical conditions. By constructing a 2D planar lattice system with discrete spin variables representing grain topological orientations, this model successfully reproduced the recrystallization topological evolution dominated by grain boundary migration.

Radhakrishnan [42] innovatively established a multiscale coupling framework that integrated macroscopic finite element analysis of cold-rolling deformation fields with mesoscopic Monte Carlo simulations of static recrystallization (SRX) kinetics in face-centered cubic (fcc) metals. Their work quantitatively correlated spatial distributions of deformation-stored energy with spatiotemporal evolution of recrystallization nuclei. By introducing a texture-sensitive anisotropic grain boundary mobility function, they accurately predicted the evolution trajectory of recrystallization texture components during annealing. The simulated microstructural characteristic parameters showed significant correlation (R^2^ > 0.91) with synchrotron radiation EBSD experimental data, establishing a paradigm for cross-scale simulation of metal recrystallization under multiphysics coupling.

21st Century Advances in Monte Carlo Multiscale Modeling by Chia-Yi Yang et al. [43] combined 3D MC modeling with reversible polymerization theory (the Shih–Aksay–Kikuchi model) to precisely predict colloidal nucleation-growth kinetics, achieving <5% error in simulated grain boundary curvature distributions compared to experiments. Okuda et al. [44] revealed the Zener parameter dominated grain morphology evolution mechanism in steel recrystallization, clarifying the critical regulation of second-phase particle distribution on abnormal grain growth.

Chun’s team [45], through combined cold-rolled pure titanium experiments and MC simulations, first demonstrated that static recrystallization grain sizes follow a heterogeneous nucleation-dominated log-normal distribution. They established a quantitative correlation model between dislocation density field and MC transition probabilities, revealing an 18% increase in recrystallization activation energy due to grain nucleus interlocking in high-strain regions. During 2006–2009, Kazeminezhad et al. [46] developed a FEM-MCM cross-scale model predicting copper wire annealing grain distributions (<8% error) through cold deformation stored energy field mapping (ΔG = 0.5ρμb^2^). Their subsequent work [47] created a universal MC model based on hardness parameters (HV = Kσ_y_^n^), enabling unified prediction of recrystallization microstructures across deformation modes (R^2^ = 0.89). Sepehrband et al. [48] developed a 3D MC model coupling recrystallization–precipitation in aluminum alloys, revealing the dynamic competition mechanism between precipitate coarsening (Ostwald index *n* = 3.1) and recrystallization growth, with 92% consistency between simulations and in situ TEM data.

Post-2010, MC methods have been extended to simulate authentic 3D grain structures, including grain shape, size, and boundary properties. Current research focuses on elucidating the influence of crystallographic orientation on texture evolution during recrystallization kinetics.

From 2014 to 2024, Monte Carlo (MC) methods continued to expand their modeling capabilities in multiphase material systems. Wang et al. [49] developed a 3D recrystallization MC model for nickel-based superalloys by introducing an anisotropic grain boundary energy function, enabling quantitative analysis of the formation mechanism of annealing twin orientation relationships ({111}<112>). Their work revealed an exponential decay relationship between abnormal grain growth direction and Σ3Σ3 twin boundary density.

**Figure 3:**
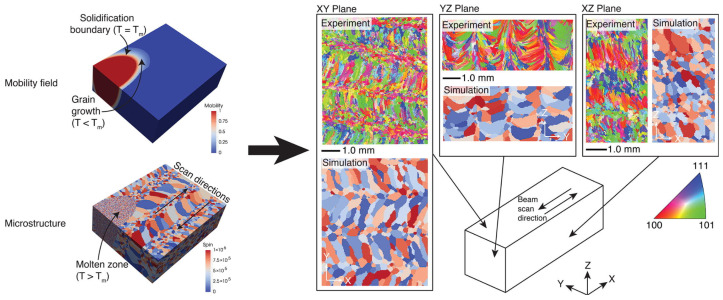
The MC method simulates additive manufacturing [35].

Zheng’s team [50] extended MC simulations beyond traditional metallic systems to polymer/gas permeation systems. By constructing a free-volume probability distribution function, they achieved diffusion coefficient predictions for pure H22S in PE/PVDF membranes with <5% error, uncovering cross-scale correlations between crystallinity and permeation flux. As shown in Figure 3, the MC method simulates additive manufacturing [51].

The core strength of MC methods lies in their dynamic probabilistic sampling capability based on the Metropolis algorithm, which efficiently couples thermodynamic driving forces (ΔGΔG) with kinetic path entropy. This makes them particularly suited for addressing complex mesoscale evolution challenges, such as spatial heterogeneity in recrystallization nucleation (Cv > 0.3Cv > 0.3) and many-body interactions between grain boundaries and precipitates.

Overall, MC is advantageous for statistically describing grain growth, recrystallization and abnormal grain growth with relatively low computational cost. Nevertheless, its physical accuracy is constrained by lattice discretization, empirical transition probabilities and the difficulty of converting Monte Carlo steps into real time. Therefore, MC is more appropriate for mesoscale topology evolution than for direct field prediction of stress, strain or solute redistribution.

### 3.2. Cellular Automata Method

The cellular automaton (CA) method is a dynamical system discrete in both time and space, capable of simulating the evolution of complex systems under discrete spatiotemporal conditions [52,53]. As a mesoscopic-level numerical simulation approach, it combines simple transition rules with mechanisms for grain evolution. A CA model typically comprises five elements: cells, cell states, cell space, neighborhood types, and transition rules [54,55,56].

**Figure 4:**
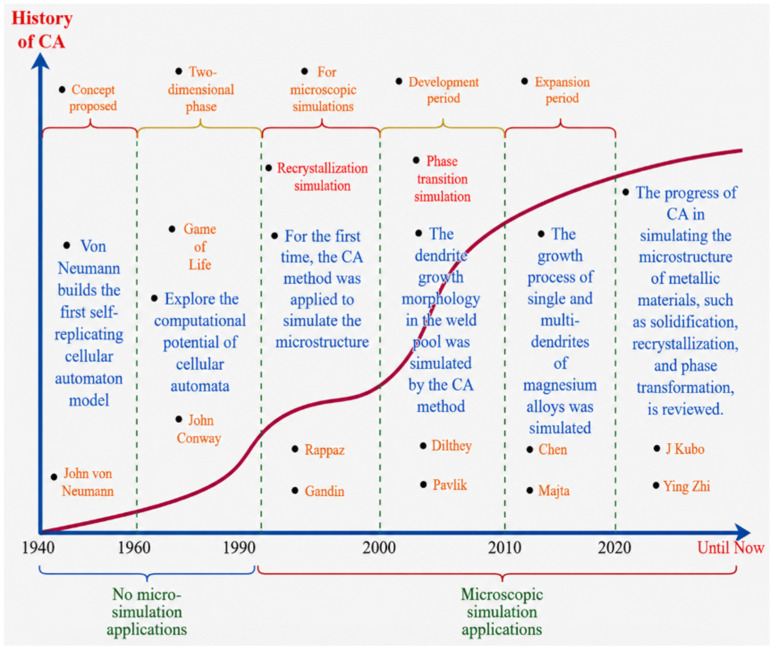
The development of the CA method.

Since the late 1990s, the Cellular Automaton (CA) method has emerged as a powerful tool for simulating metallic microstructures. As a discrete model, CA couples temporal and spatial elements with transition rules to represent continuous processes. It replaces complex partial differential equations with discrete interactions of state variables between neighboring elements, making it an efficient computational framework [57]. When applied to recrystallization simulations, CA integrates nucleation models and grain growth models—both temperature- and strain-rate-dependent—to characterize microstructural evolution under varying forming conditions. By assigning values to state variables at each cell, the distribution of grains within the simulated domain can be explicitly described. These cell state variables evolve spatially and temporally according to predefined rules, enabling the simulation of time- and space-resolved microstructural transformations [58]. Figure 4 shows the development process of the CA method [59,60,61,62,63,64].

The numerical implementation of a CA model follows these key steps:

(1) Define the cellular space: Discretize the simulation domain into a grid of cells, where each cell represents a microscopic region of the material.
(2) Initialize cell states: Assign initial states to each cell based on predefined conditions (e.g., temperature, composition distribution).
(3) Establish local rules: Formulate state transition rules governed by physical mechanisms (e.g., solidification, recrystallization).
(4) Iterative computation: Update cell states incrementally over discrete time steps to simulate microstructural evolution.
(5) Data extraction and analysis: Track critical metrics (e.g., grain size, morphology, spatial distribution) during the simulation and correlate them with material properties.

The cellular automaton (CA) method for simulating metal recrystallization has evolved significantly through key developments: Marx et al. [65] developed the first 3D CA model in 1999, integrating crystallographic texture, deformation substructures, and temperature fields to achieve multiscale simulation of recovery, nucleation, and growth during the static recrystallization of cold-worked metals, while pioneering quantitative characterization of microstructural heterogeneity. Janssens’ team [66] subsequently proposed a 3D stochastic grid CA model that established quantitative correlations between spatiotemporal evolution and grain boundary dynamics through migration theory.

To address dynamic recrystallization (DRX) challenges, Yazdipour [67] constructed an irregular grid-based CA framework in 2008, successfully predicting pure copper’s DRX microstructural evolution and macroscopic flow behavior using dislocation density–thermodynamics coupling models. Hallberg et al. [68] further advanced 3D probabilistic CA modeling in 2010 with stochastic nucleation and grain boundary migration rules, achieving the first quantitative description of non-steady-state grain evolution during dynamic discontinuous recrystallization.

**Figure 5:**
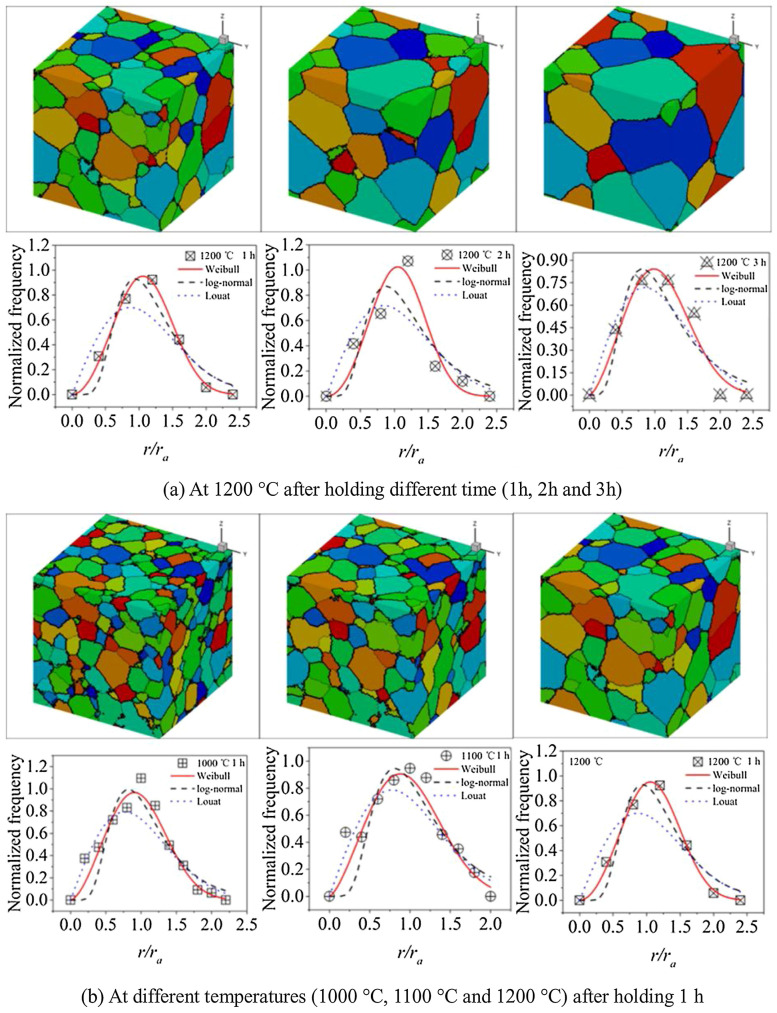
Grain size simulation comparison results simulated by the CA method [72].

CA applications expanded to complex material systems and processing scenarios: Liu et al. [69] innovatively coupled the LJ dislocation density model with CA in 2013, enabling cross-scale analysis of AZ31 magnesium alloy’s DRX behavior during hot compression while revealing temperature/strain-rate effects on nucleation mechanisms and dislocation redistribution. Popova’s team [70] developed a multi-field coupled CA model in 2014 for thermomechanical processing, breaking traditional simulation limitations by quantifying texture evolution–DRX kinetics relationships. In Figure 5, the grain size simulation results simulated by the CA method are compared [71].

Recent advancements continue pushing CA’s boundaries: Li et al. [72] extended CA to magnesium alloy slip behavior modeling, elucidating AZ80’s microstructural response through coordinated dislocation slip activation and grain boundary migration. Su et al. [72] created a 3D CA model for Fe-1C-1.5Cr steel austenite coarsening, integrating carbon/chromium diffusion kinetics and austenite boundary migration rules to achieve quantitative phase transformation predictions, establishing new paradigms for alloy heat treatment optimization. These developments demonstrate CA’s expansion from fundamental recrystallization studies to multi-material processing simulations.

The cellular automaton (CA) method for metallic recrystallization simulations achieved significant technical breakthroughs and deepened mechanistic understanding in 2021. Liu et al. [73] proposed an innovative probabilistic CA two-step strategy, developing a universal microstructure generation framework through an equiaxed grain–subgrain composite structure algorithm. Their MATLAB (R2021a)-based prediction model for substructures in 2219 aluminum alloy during thermomechanical processing first revealed quantitative control mechanisms of deformation temperature/rate on the dynamic transformation pathways of low-angle grain boundaries (LAGBs). Meanwhile, Kugler’s team [74] focused on static recrystallization, establishing correlations between initial microstructure topological parameters and recrystallization kinetics, which elucidated how grain boundary network geometry regulates recrystallization activation energy and abnormal grain growth.

**Figure 6:**
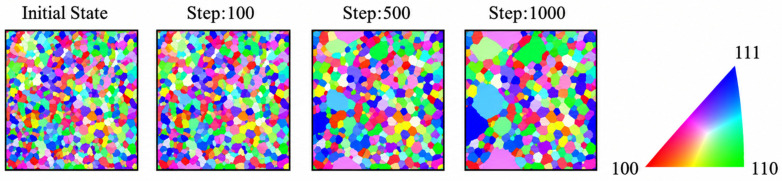
Simulation comparison chart of different cell counts [75].

In grain evolution dynamics, Murata, K et al. [75] innovatively integrated CA with classical parabolic grain coarsening laws. By constructing dynamic models of grain size-topology distribution, they achieved multiscale characterization of curvature-driven grain boundary migration during steady-state coarsening. Current research confirms CA’s core strength lies in efficiently reconstructing spatiotemporal evolution features of complex systems through localized interaction rules, particularly excelling in simulating non-equilibrium phase transformations like dynamic recrystallization and slip band formation. However, its discretized nature still faces theoretical limitations in precisely representing continuous field variables and convergence challenges when handling multi-field coupling boundary conditions and complex geometric morphologies [76]. Future advancements require breakthroughs in continuous–discrete coupling modeling techniques and physics-informed intelligent evolution rule design. Figure 6 shows the simulation comparison chart of different cell numbers [75].

CA provides one of the best balances between computational efficiency and microstructure morphology prediction. It is particularly useful for large-domain simulations of recrystallization and solidification. However, CA results depend strongly on cell size, neighborhood definition and transition rules, which may reduce transferability between different alloys and processing conditions. Thus, CA is most reliable when its rules are calibrated by experiments or coupled with thermodynamic or kinetic models.

### 3.3. Vertex Model

The vertex model is a computational tool used for simulating the evolution of the microstructure of metals. By idealizing the material as a continuum composed of grain boundary segments and vertices, it can effectively describe the changes in the microstructure such as the growth of grains, the migration of grain boundaries, and recrystallization. In the microscopic simulation of metals, the vertex model is widely applied to the simulation of the recrystallization process (including dynamic recrystallization and static recrystallization), grain growth, and the evolution of multiphase microstructures [77]. It can be coupled with other models such as the crystal plasticity model and the cellular automaton model to form a multiscale simulation framework, thus enabling a more comprehensive description of the changes in the microstructure.

The vertex model was initially proposed to describe the microstructure of solid materials. It idealizes solid materials or soap bubble-like structures as a homogeneous continuum of grain boundary segments that intersect and connect with each other at the vertices (i.e., at the grain boundary junctions). The theoretical basis of this model provides an important framework for subsequent microscopic simulations [78,79,80,81].

From the end of the 20th century to the beginning of the 21st century, the vertex model began to be applied to the simulation of dynamic recrystallization (DRX). Dynamic recrystallization is an important softening mechanism of metals during thermal processing, and its numerical simulation methods have been widely studied. By describing the growth of grains and the migration of grain boundaries, the vertex model can effectively simulate the evolution of the microstructure during the recrystallization process [82].

In recent years, with the development of multiscale simulation methods, significant progress has also been made in the coupled application of the vertex model with the crystal plasticity model and the precipitation model.

The core of the vertex model is based on the principle of minimizing the grain boundary energy and the kinetic evolution equation, and its mathematical expressions are as follows:

(1) The formula for the total energy of the system

$$E=\sum_{\mathrm{edges}}\;\gamma_{\mathrm{ij}}L_{\mathrm{ij}}+\sum_{\mathrm{vertices}}\;\alpha \kappa^{2}$$

In the equation, the first term *γ_ij_* represents the interface energy density of the grain boundary *i*-*j*, and *L_ij_* corresponds to the edge length, reflecting the contribution of the grain boundary length to the energy. The second term *κ* is the local curvature of the grain boundary, and *α* is the curvature stiffness coefficient, which is used to characterize the Gibbs-Thomson effect.

(2) Vertex dynamics equations

The vertex motion is driven by an energy gradient and is described as an overdamped dynamic:

$$v_{k}=M\cdot \nabla_{r_{k}}E$$

In the equation, *v_k_* represents the migration velocity of vertex *k*, *M* is the grain boundary mobility (which is related to temperature and grain boundary type), and $\nabla_{r_{k}}E$ is the gradient of the total energy with respect to the position *r_k_* of the vertex, characterizing the mechanical equilibrium state of the grain boundary network.

(3) Curvature driving mechanism

The velocity of grain boundary migration is positively correlated with the local curvature (curvature flow law):

$$v_{n}=M\gamma \kappa$$

In the equation, *n* represents the normal velocity, *γ* is the average interface energy, and *κ* = R_1_ is the curvature. In the vertex model, curvature is indirectly calculated through the geometric angles of adjacent edges, driving the adjustment of vertex positions to reduce the system energy. The numerical simulation of the vertex model is implemented through the following structured process, covering geometric modeling, dynamic evolution, and topological adaptive control:

(1) Geometric modeling and initialization

Grain boundary network discretization: The polycrystalline system is abstracted into an unstructured mesh composed of vertices and edges. In the two-dimensional model, polygons are used to represent grains, while the three-dimensional extension requires defining the topology of grain boundary surfaces and edges. Parameter setting: the interface energy density *γ_ij_*, grain boundary mobility *M*, and curvature stiffness coefficient *α* are assigned based on material properties and need to be calibrated through molecular dynamics simulations or experimental data.

(2) Dynamic evolution iteration

Energy gradient calculation: For each vertex *k*, calculate the negative gradient of the total energy of the system with respect to its coordinates, *F_k_* = −$\nabla_{r_{k}}E$, where the energy terms include the contributions from adjacent edge lengths ∑*γ_ij_L_ij_* and curvature energy terms ∑*ακ*^2^.

Vertex motion update: An explicit time integration method (such as the forward Euler method) is used to update the vertex positions according to *v_k_* = *MF_k_*. The time step Δ*t* must satisfy numerical stability conditions.

(3) Topological adaptive adjustment

T1 process (two-dimensional topological reconstruction): When the distance between adjacent vertices is less than a critical threshold, a reconnection mechanism of four vertices is triggered, simulating topological mutations caused by grain boundary sliding or grain merging.

(4) Boundary condition handling

Periodic boundary: Apply periodic extension at the boundaries of the simulation area to eliminate finite size effects, suitable for simulating macroscopically homogeneous systems. Fixed constrained boundaries: Apply position constraints to the vertices at surfaces, inclusions, or dislocation pinning regions to simulate the effects of external fields or defects on the suppression of grain boundary migration.

(5) Termination conditions and microstructure quantification

Simulation termination criteria: Set a maximum simulation time tmax, or determine that the system has reached a quasi-steady state when the energy change rate ∣Δ*E*/*E*∣< 10^−5^.

K. Piękoś and B. Bacroix et al. [83] were the first to extend the vertex model to encompass the entire process of recrystallization during the annealing of cold-rolled polycrystalline copper. Y. Mellbin and M. Ristinmaa [84] developed a model that combines a crystal plasticity model with a graph-based vertex model, capable of simultaneously describing finite strain deformation, texture evolution, and microstructural evolution. By integrating the crystal plasticity model with the graph-based vertex model, they successfully simulated the dynamic recrystallization process of pure copper under high-temperature large deformation conditions. Cameron McElfresh and Jaime Marian [85] studied the microstructural evolution of cold-rolled iron during the static recrystallization (SRX) process, developing a comprehensive physical model based on coupled crystal plasticity and grain boundary dynamics. This model combines finite element polycrystalline plasticity simulations with a two-dimensional vertex dynamics approach to explore a large parameter space affecting static recrystallization.

The advantage of the vertex model lies in its ability to accurately describe the geometric shape of grains and the migration behavior of grain boundaries, making it particularly suitable for simulating grain growth and phase transformations during recrystallization processes. However, its disadvantages include high computational complexity, especially when dealing with large-scale three-dimensional problems, resulting in lower efficiency. Additionally, the establishment and solution processes of the model are relatively complex and sensitive to initial conditions and parameter settings.

The vertex model has high geometric fidelity in describing grain boundary networks and topological transitions. Its limitation lies in the increasing complexity of three-dimensional implementation and the sensitivity of topological operations to numerical parameters. Therefore, it is suitable for mechanistic studies of grain boundary migration, but less efficient for large-scale industrial process simulations.

## 4. Continuous Field Model

### 4.1. Artificial Neural Network (ANN)

Artificial Neural Networks (ANNs) have emerged as a mathematical model in recent years, characterized by features such as error feedback, error correction, and tracking learning when using multilayer neural networks. This is particularly advantageous for addressing nonlinear and complex issues, such as dynamic recrystallization [86]. ANNs are widely applied in fields like pattern recognition, classification, and regression prediction. In materials science, ANNs can be used to analyze the microstructure of grains to predict material properties or optimize fabrication processes. Common neural network architectures include feedforward neural networks, convolutional neural networks (CNNs), and recurrent neural networks (RNNs). The fundamental equations of ANNs primarily involve the activation process of neurons, forward propagation, and backward propagation [87]. For grain microstructure analysis, CNNs are often a better choice because they can effectively process image data and extract features from images.

In 1943, Warren McCulloch and Walter Pitts proposed a mathematical model simulating neurons, marking the birth of artificial neural network theory. In 1958, Frank Rosenblatt laid the foundation for the development of multilayer neural networks. During this period, the application of ANNs focused mainly on simple classification and regression tasks, without involving specific three-dimensional micro-simulations [88]. Entering the 21st century, with improvements in computational power and data storage capacity, ANNs began to be used for processing high-dimensional data, such as electron microscope images and EBSD data. In grain microstructure research, ANNs have been applied for image classification and feature extraction. In recent years, the rise of deep learning has ushered ANNs into the era of multilayer deep networks, with models like CNNs being utilized for complex image processing tasks. In materials science, ANNs are no longer limited to performance prediction but have gradually become important tools in three-dimensional micro-simulation.

For three-dimensional grain reconstruction, CNNs are used to process microscopic images and reconstruct the three-dimensional microstructure of metallic materials. For grain boundary behavior analysis, deep learning is leveraged to predict grain boundary migration behavior and its impact on microstructural evolution. Generative Adversarial Networks (GANs) are employed to simulate the growth process of grains, generating three-dimensional structures consistent with experimental results. In the past decade, ANN methods have been applied to the microstructural simulation of metals.

The team led by Cai Zhongman [89] developed a multitask framework using ANNs, successfully predicting the grain size during the dynamic recrystallization of heat-resistant steel (error < 6.3%) and constructing a hot processing map. Hashemi et al. [90] utilized ANNs to establish a multiscale model for the static recrystallization of polycrystalline materials, achieving a correlation coefficient of 0.91 with EBSD experimental results. Addressing parameter identification challenges, Lou et al. [91] combined BP neural networks with genetic algorithms to optimize the constitutive model parameters of magnesium alloys, significantly improving prediction accuracy (R^2^ > 0.98). In the field of process modeling, Seyed Salehi et al. [92] proposed an ANN training algorithm based on differential datasets to achieve thermomechanical coupling simulation of the hot rolling process, with a rolling force prediction error controlled within ±4.5%. The application of ANNs has extended to atomic-scale simulations: Irie et al. [93] constructed a machine learning potential function for metallic sodium, accurately reproducing its melting-crystal phase transition process (energy error < 1.5 meV/atom). The ANN-crystal plasticity finite element coupling model developed by Ali et al. [94] can predict the deformation texture of aluminum alloys in real-time (stress error < 5.2%). In addition, Qin et al. [86] established a global rheological stress model of magnesium alloy through ANNs, which achieved high-precision mapping over a wide range of temperature regions and strain rates (R^2^ = 0.992).

**Figure 7:**
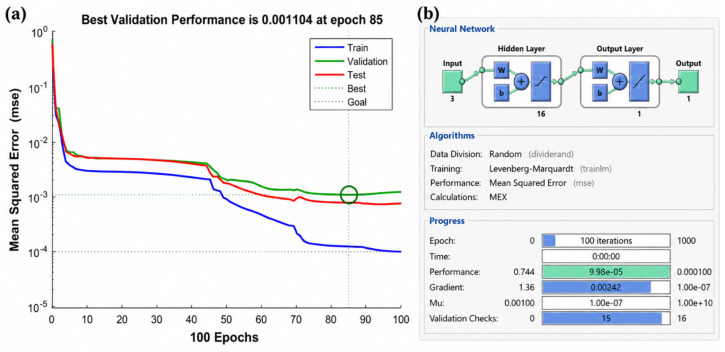
Performance evaluation and architecture of the ANN model. (**a**) Mean squared error (MSE) curves for the training, validation, and test datasets, with the best validation performance obtained at epoch 85. (**b**) Schematic architecture and training progress of the ANN model [89].

As shown in Figure 7, ANN-based prediction can be used to evaluate dynamic recrystallization behavior and processing response [89].

Yan et al. [95] investigated the hot compressive flow behavior of an Al–6.2Zn–0.70Mg–0.30Mn–0.17Zr alloy and developed both Arrhenius constitutive and ANN models to predict flow stress, with the ANN model showing higher prediction accuracy. Tong Wang and Yongzheng Chen et al. [96] studied the design of novel AZE311 and AZX311 magnesium alloys by adding 1% gadolinium (Gd) and calcium (Ca) to AZ31 alloy to improve its high-temperature mechanical properties. They introduced a dimensionless correction factor λ based on Artificial Neural Networks (ANNs) to enhance these equations, significantly improving the predictive accuracy of the model. Quan Li [97] and his team developed a high-temperature constitutive model for AZ61 magnesium alloy using a backpropagation neural network (BP network) and trained the network with experimental data, making it a knowledge-based constitutive relationship model. Jie Yan and Wen-bo Song et al. [97] investigated the hot compression rheological behavior of Al-6.2Zn-0.70Mg-0.30Mn-0.17Zr alloy at deformation temperatures of 623 to 773 K and strain rates of 0.01 to 20 s^−1^. They established an Arrhenius constitutive equation and an ANN model to predict the alloy’s rheological behavior. Nie, K. et al. [98] studied the hot deformation behavior of AZ91 magnesium alloy. Their research analyzed the hot compression tests of AZ91 alloy using the Arrhenius model and backpropagation artificial neural network (BP-ANN) methods, with tests conducted in the temperature range of 473–623 K and strain rates of 0.001–1 s^−1^.

**Figure 8:**
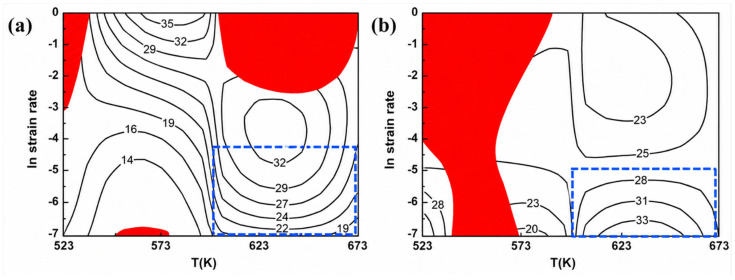
Comparison of processing maps constructed under different modeling conditions. (**a**) Processing map I; (**b**) processing map II. The red shaded regions represent flow instability zones, and the blue dashed rectangles denote the suitable processing domains with relatively stable deformation behavior [98].

Liu and Jin [99] quantified the influence of twinning effects on the flow stress of Mg-3Sn-1Mn alloy for the first time by constructing a feedforward-backpropagation artificial neural network (Feedforward-BP ANN), revealing a contribution of 17.3%. Their model achieved a prediction error of less than 8.5% within the strain rate range of 0.001–1 s^−1^. Cheng and Ding [100] compared the stress prediction accuracy of a BP deep neural network (BP-DNN) in their study of the ZA270.15Ce alloy, finding that it improved by 12.7% (R^2^ = 0.986) over a conventional modified Arrhenius model, demonstrating the advantages of neural networks in constitutive modeling of rare earth element-doped alloys. Sabokpa and Haghdadi [101] systematically evaluated the applicability of the Arrhenius equation and an ANN for AZ81 magnesium alloy, confirming that the ANN model exhibited superior prediction stability in the dynamic recrystallization dominant region (temperature > 350 °C), with a reduction in standard deviation by 42%. For modeling high-temperature rheological behavior, Talebi Anaraki and Akbarzadeh [102] proposed a combined modeling framework using inverse methods and an ANN, successfully decoupling the competition between work hardening and dynamic softening mechanisms of AZ61 magnesium alloy in the temperature range of 300–450 °C, with relative error control of peak stress predictions within ±3.2%. Figure 8 shows the microstructure of the as-cast AZ91 magnesium alloy simulated by the ANN method [98].

ANN-based models are powerful for rapid prediction and nonlinear mapping once sufficient training data are available. Their main weakness is that prediction accuracy may decrease significantly outside the training domain, and the physical interpretability of learned features remains limited. Therefore, an ANN is more suitable as a surrogate model, acceleration tool or process-optimization model rather than a standalone physics model.

### 4.2. Phase Field Method

The Phase Field (PF) model is established on the basis of thermodynamics, by taking into account the combined effect of the ordering potential and the thermodynamic driving force, and establishing the PF equation to describe the dynamics of the system evolution. Compared with the Monte Carlo (MC) method, the greatest advantage of the phase field method is that it can avoid the trouble of tracking the interface of the microstructure, and can accurately reproduce the movement of the interface and the morphology of the microstructure. Its idea is to introduce one or more continuous field variables, and use the diffuse interface model to replace the traditional sharp interface model to describe the interface of the microstructure. In the phase field model, various thermodynamic driving forces related to the evolution of the microstructure can be conveniently considered, including crystal energy, interfacial energy, elastic strain energy, chemical energy, magnetic field energy, etc. [103,104,105]. The core idea of the phase field method is to describe the evolution process of the microstructure by introducing phase field variables and based on the free energy functional, and obtain the numerical solutions of the phase field variables through numerical solution methods.

The phase field method has the following advantages: it can naturally handle complex microstructures and phase interfaces; it can be coupled with other physical fields; and it is suitable for the simulation of a variety of materials and physical processes. The phase field method also has some challenges, such as a large amount of calculation and the need to reasonably select model parameters. In 1958, John W. Cahn and John E. Hilliard proposed the classic method in the “phase field theory”, but the earliest application of the numerical phase field method appeared in 1970. In 1980, scholars such as Kurt Binder and Wolfhard Janke further developed the numerical methods of the phase field method, especially using the finite difference method and the finite element method to solve the Cahn–Hilliard equation, thus enabling large-scale simulations on a computer. 

Based on the description of thermodynamics and the diffuse interface, the PF model has been proven to be very powerful in capturing the evolution of the microstructure without the need to explicitly track the interface as in the traditional sharp interface model. The PF model can simulate phenomena such as recrystallization, grain growth, phase transformation, and solidification, which have been widely developed in the past two decades. In the PF model, the microstructure is represented by a set of phase field variables (order parameters) *η*, which are continuous functions of the space r and time t.

In the PF model, order parameters are crucial for describing the microstructural evolution of materials, as they represent the state of the material in space and time. To analyze the evolution of these order parameters, it is typically necessary to establish time-dependent partial differential equations based on thermodynamic principles. For conserved order parameters (such as concentration in nonlinear diffusion), the most common form of these equations is the Cahn–Hilliard equation. For non-conserved order parameters (such as phase or grain orientation), the Allen–Cahn equation is used.

$$\frac{\partial \eta_{i}(r,\;t)}{\partial t}=-L_{ij}\frac{\partial F}{\partial \eta_{j}}\;i,\;j=1,\;2,\;\cdots ,\;n$$

In this context, *M_ij_* represents time; *L_ij_* denotes the diffusion rates of different conserved variables; *F* refers to the mobilities of different non-conserved variables; and *F* is the free energy functional of the system. The application of the phase field (PF) method in various phenomena is primarily reflected in the description of the free energy functional. In the simulation of dynamic recrystallization (DRX), the Allen–Cahn equation is typically employed.

$$F_{tot}=F_{gb}+F_{def}=\int_{V}\;f_{gb}dV+\int_{V}\;f_{def}dV$$

In this context, *f_gb_* and *f_def_* represent the grain boundary (gradient) energy density and the deformation energy density, respectively. *V* denotes the simulation domain. Researchers have proposed different formulas to describe these two types of energy densities for dynamic recrystallization (DRX) simulations.

In 1990, Liu et al. [106] developed a three-dimensional phase field model (PFM) parallel algorithm that improved simulation efficiency by approximately 40%, successfully applying it to simulations of metal solidification and grain boundary migration. In 2006, Böttger introduced a multi-component multi-phase field model, which efficiently simulated the solidification process of alloys such as AZ31 by coupling thermodynamic databases with a random nucleation algorithm, reducing computation time by 65% [107]. In 2009, Fries et al. [108] further developed the MCMPF method by introducing a thermosolutal diffusion coupling algorithm, which accurately predicted the influence of cooling rates on the grain structure of aluminum alloys with an error of less than 12%.

In the area of solid-state phase transformation simulations, C. Liu et al. [109] coupled crystal plasticity constitutive models with phase field dynamics, establishing a comprehensive prediction model for twin nucleation, expansion, and growth, achieving an 89% match with experimental data. Wang et al. [110] systematically developed a multi-model PFM framework for magnesium alloys covering the entire process of solidification, recrystallization, and solid-state phase transformation, achieving a correlation coefficient of 0.93 between the twin evolution model and experimental data. Kunok Chang and Heebaek Chang employed a multi-parameter grain growth model and implemented OpenMP to enhance computational efficiency, performing phase field simulations of isotropic and anisotropic grain growth in both two-dimensional and three-dimensional systems [111]. Their research utilized desktop workstations for large-scale 2D and 3D phase field grain growth simulations, demonstrating the impact of anisotropic grain boundary energy on microstructural features, including grain aggregation phenomena and grain size distribution.

For high-temperature alloys, the team led by Lin Yongcheng coupled PFM with cellular automata (CA) to establish a multi-scale predictive model for the dynamic/subdynamic recrystallization of nickel-based alloys, optimizing heat treatment processes to reduce the degree of microstructural mixing by 40% [112].

**Figure 9:**
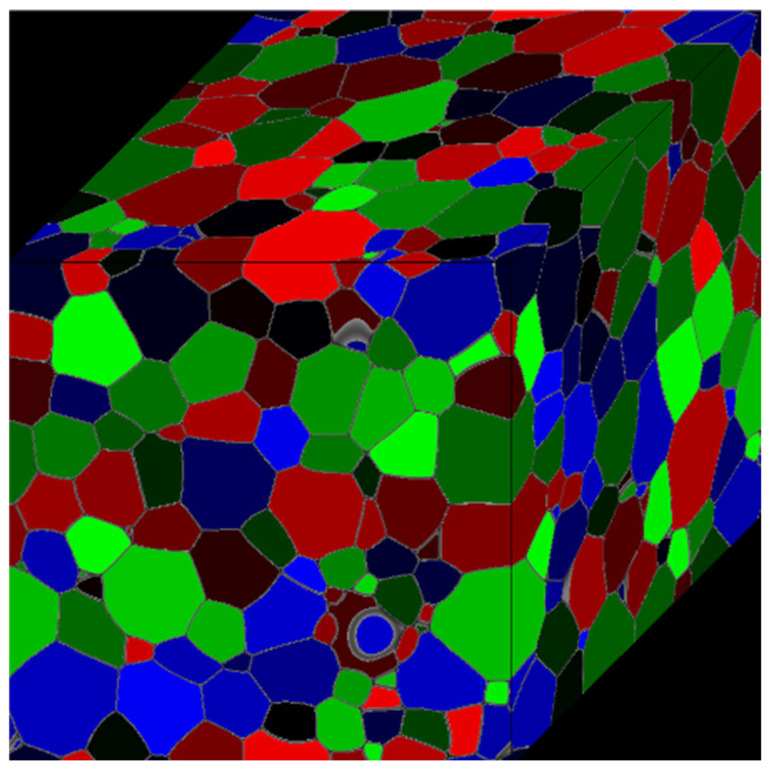
The grain growth structure simulated by the phase field method [111].

PFM technology is rapidly penetrating complex engineering problems. The PF-DDRX-SP model proposed by Zhu et al. [113] successfully predicted the uniformity of DRX grains in thermomechanical processing (with a coefficient of variation of less than 15%) by quantifying the coupling effects of second-phase particle pinning and nucleation mechanisms. Erden Yildizdag innovatively combined PFM with the material point method (MPM) to develop a cross-scale simulation framework for melting and solidification aimed at 3D printing, achieving sub-micron resolution (with an error of less than 2.1 μm) in metallic additive manufacturing simulations through a free surface tracking algorithm [114]. Figure 9 shows the grain growth structure simulated by the phase field method [111].

The phase field method (PFM) has significant advantages and disadvantages in the microstructural simulation of metals. Its advantages mainly lie in its ability to accurately simulate the microstructural evolution of materials, such as dendritic growth, phase transitions, and microsegregation. PFM describes the evolution of the phase field in space through mathematical models, capturing the complex changes in microstructures, thereby providing a theoretical basis for material design and performance optimization. However, PFM also has some shortcomings. The grain boundary width in the PF model is merely a numerical parameter and is not determined through physical methods, so caution is needed when setting the grain boundary width to ensure proper partitioning of the interface. Additionally, PFM requires a significant amount of computational resources, especially for three-dimensional simulations, which can be time-consuming. The establishment of the model demands accurate material parameters, and acquiring these parameters can often be challenging. Furthermore, PFM may overlook some complex factors present in actual physical processes, such as the effects of flow fields and temperature fields, which can lead to discrepancies between simulation results and real-world conditions.

PF has high accuracy for interface-controlled evolution because it avoids explicit interface tracking and naturally handles complex morphology. However, its computational cost is high, especially in three-dimensional multi-component and multi-phase systems. The diffuse interface width and mobility parameters also require careful calibration. Therefore, PF is preferred when interface morphology and thermodynamic consistency are more important than computational efficiency.

### 4.3. Finite Element Method

The Finite Element Method (FEM) is based on the idea of discretizing a continuous geometric entity into a finite number of elements, and setting a finite number of nodes on each element. It is like regarding the continuous geometric entity as a group of elements connected by nodes [115]. In the context of dynamic recrystallization, it means discretizing the whole hot-deformed alloy into a finite number of elements and setting a finite number of nodes in each element. During the hot-deformation process of the alloy, the dynamic recrystallization is a process where changes initiated at the nodes drive changes in the elements. When these changes are visualized through relevant software, it is the finite element (FE) simulation of the alloy’s dynamic recrystallization [116].

The original concept of the FEM can be traced back to the 1940s, first proposed by R. Courant. In 1943, Courant used the finite difference method to solve problems in elastic mechanics for the first time, laying the foundation for the FEM. Scholars like J. Hrennikoff and O. C. Zienkiewicz further developed this method in the fields of engineering mechanics and structural analysis respectively. In particular, Zienkiewicz presented the mathematical basis of the finite element method in the 1960s and promoted its application. From the 1970s to the 1980s, the FEM gradually spread to various engineering and physical problems, especially its application in complex three-dimensional problems gained attention.

**Figure 10:**
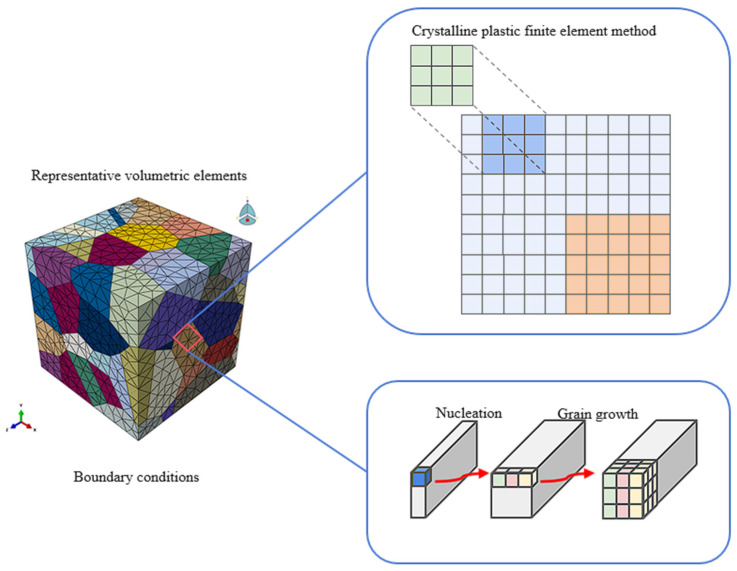
Schematic diagram of the multiscale microstructure simulation framework. The representative volume element (RVE) with boundary conditions is coupled with the crystal plasticity finite element method (CPFEM), while nucleation and grain growth are introduced to describe the evolution of the microstructure.

The main tools for applying the FEM in the simulation of metal microstructures include software such as DEFORM, ProCAST, QForm, Simufact, and LS-DYNA. DEFORM can simulate the evolution of the microstructure during metal forming and heat-treatment processes and is suitable for processes like forging and rolling. ProCAST is mainly used for casting process simulation and can predict the microstructure and properties of castings. QForm focuses on metal forming processes and can simulate the changes in the microstructure during forging and rolling. Simufact is suitable for multi-step forming processes and can predict the influence of the microstructure on material properties. LS-DYNA, with its multi-physical field coupling ability, can accurately simulate complex forming processes. Figure 10 shows the framework of the finite element method for crystal plasticity.

The Crystal Plasticity Finite Element Method (CPFEM) is an advanced method developed in recent years. It can take into account the influence of grain morphology and orientation on macroscopic mechanical properties. By establishing a three-dimensional Voronoi model, it can simulate the evolution of the microstructure of polycrystalline materials under finite deformation. This method can effectively predict the anisotropic behavior of materials and provide reliable mechanical property predictions for practical engineering applications.

Ji et al. [117] combined EBSD characterization with full-field crystal plasticity finite element simulation to reveal the texture gradient, grain-orientation rotation, shear-band-related deformation, and orientation-dependent recrystallization behavior of CVD tungsten during multi-step rolling. Musienko et al. [118] developed a real three-dimensional crystal-plasticity finite element model of an OFHC copper polycrystal under monotonic tension and compared the simulated stress–strain response, local strain fields, lattice rotation, and slip-system activity with experimental measurements.

**Figure 11:**
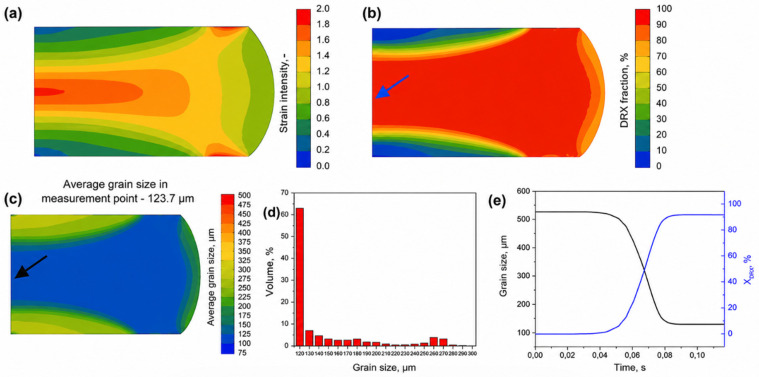
Simulation results of strain distribution, dynamic recrystallization, and grain size evolution during hot deformation. (**a**) Strain intensity distribution; (**b**) dynamic recrystallization (DRX) fraction distribution; (**c**) average grain size distribution, with the average grain size at the selected measurement point being 123.7 μm; (**d**) grain size distribution histogram; (**e**) evolution of grain size and DRX fraction with deformation time [121].

Bong et al. [119] developed a crystal plasticity finite element (CPFE)–Marciniak–Kuczynski (M-K) coupling model, which successfully predicted the forming limit strain of magnesium alloy sheets (with an error of less than 8%) and quantified the competitive relationship between the slip and twinning mechanisms. Li and Cheng [120] proposed a FEM modeling method based on microstructural characteristics, revealing the quantitative relationship between the dynamic compression properties and microstructure evolution of duplex titanium alloys (R^2^ > 0.95). In the field of high-temperature deformation, Zyguła [121] constructed a kinetic model of grain growth and dynamic recrystallization covering the temperature range of 900–1250 °C through FEM, and the prediction accuracy of its critical strain reached ±5%. Figure 11 shows the hot pressing test diagram and grain length curve using the finite element method [121]. In recent years’ research, Panda et al. [122] established a coupling framework of macroscopic FEM–microscopic phase field (PF), achieving the full-process prediction of the thermal-microstructure evolution of AlSi10Mg alloy during the additive manufacturing process (with a 40% improvement in computational efficiency). Facing the challenges of insufficient core deformation and coarse grains during the rolling process of ultra-thick steel plates, the research team led by Xu Chen and Cai Qingwu from the University of Science and Technology Beijing developed a multi-field coupling model of thermal, mechanical, and microstructural behaviors based on the secondary development of Abaqus. The team used Python 3.6.5 scripts and the FORTRAN subroutine VUSDFLD to expand the functions of Abaqus, and combined the Johnson–Cook material deformation model with the recrystallization kinetic equations to simulate the dynamic and static recrystallization behaviors during the rolling process [123].

FEM and CPFEM are highly suitable for deformation-driven microstructure evolution because they can incorporate complex geometry, boundary conditions and constitutive behavior. However, FEM alone cannot naturally describe grain boundary migration or phase interface evolution. For this reason, FEM is most effective when coupled with CA, PF or Level Set models in thermo-mechanical microstructure simulations.

### 4.4. Level Set Method

The level set model is a numerical technique used for interface tracking and shape modeling. It was first proposed by Osher and Sethian in 1988. Its core idea is to transform the evolution process of a curve or a surface into the evolution process of an implicit function, and describe the changes in the interface through the evolution of the implicit function. This method can perform numerical calculations on a Cartesian grid without the need to parameterize the curve or surface, thus naturally handling topological changes, such as the splitting, fusion, and reconnection of curves.

The application of the level set model in metal microstructure simulation is due to the fact that the change in its function is continuous, so it is often used in the simulation of additive manufacturing and recrystallization processes. In the field of additive manufacturing, the level set method is widely used to optimize manufacturing processes and structural designs. For example, the level set method combined with topology optimization technology can effectively handle manufacturing constraints in additive manufacturing, such as overhang angle constraints, and thus design self-supporting structures. In addition, the level set method is also used in the process planning of hybrid additive and subtractive manufacturing. Through implicit modeling and regional division, it enables the efficient processing of complex structural parts.

In terms of recrystallization process simulation, the coupling of the level set model with the crystal plasticity finite element method (CPFEM) provides a new solution for the full-field modeling of dynamic recrystallization (DRX). This coupling method can accurately describe the evolution of the microstructure during the recrystallization process, including the migration of grain boundaries and the nucleation of new grains. For example, in the recrystallization simulation of 304L steel, the level set model successfully predicted the evolution of the recrystallization fraction and was verified by comparison with experimental data.

The core of the level set method is to represent the interface through an implicit function that is one dimension higher. Assume that the interface Γ(*t*) is a curve or a surface that evolves with time t, and the level set function *ϕ*(*x*, *t*) is defined as:

$$k=\mathrm{Aexp}\left(-\frac{Q_{r}}{RT}\right)$$

Among them, *ϕ*(*x*, *t*) is a scalar function, and its zero isosurface represents the position of the interface. The applications of the level set method typically include the following steps:

(1) Initialization of the level set function: Select an appropriate initial level set function *ϕ*(*x*, 0), which is usually a signed distance function, and its zero isosurface represents the position of the initial interface.
(2) Definition of the interface movement velocity *F*: According to the specific problem, define the movement velocity *F* of the interface. This can be the velocity field in a physical process, such as the velocity field in fluid dynamics, or the phase transformation velocity in materials science.
(3) Solving the level set equation: Use numerical methods to solve the level set equation ∂*t*∂*ϕ* + *F*∣∇*ϕ*∣ = 0. Common numerical methods include the finite difference method, the finite element method, etc.
(4) Re-initialization: Regularly re-initialize the level set function to make it satisfy the condition of the signed distance function ∣∇*ϕ*∣ = 1.
(5) Post-processing: At each time step, obtain the position of the interface by extracting the zero isosurface of the level set function. This can be achieved through an isosurface extraction algorithm (such as the Marching Cubes algorithm).

In 1992, M. Munier and D. Grensing H-U [124] published a study on the exact phase transition points of the vertex model on a lattice with an even coordination number. The research background is based on the need for accurate calculation of phase transition points in statistical mechanics, especially near the phase transitions defined by critical exponents.

M. Bernacki and R. E. Loge [125] proposed a new level set framework for simulating the microstructural evolution during the primary static recrystallization process in polycrystalline materials. This framework is based on the kinetic laws of grain boundaries, links the interface velocity with the thermodynamic driving force, and has been simulated in both two and three dimensions. R. Logé and T. Coupezj [126] proposed a digital microstructure-based multiscale modeling method using the level set approach for simulating the plastic deformation of metals and the subsequent static recrystallization. It was demonstrated that the level set method can naturally handle complex topological events, such as the disappearance of grains and the nucleation of new grains. Håkan Hallberg [127] proposed a finite element model based on the level set method for simulating the microstructural evolution during the recrystallization process. This model represents grains and grain boundaries through the level set function, avoiding the generation of overlaps between grains or vacuum regions.

The level set model has significant advantages in handling complex interface tracking and shape modeling problems, especially in naturally dealing with topological changes and not requiring parameterization of the interface. However, its disadvantages, such as high computational cost, numerical stability issues, and re-initialization operations, limit its widespread use in some specific applications. In the future, the development directions of the level set model may include optimizing numerical algorithms, improving computational efficiency, and deeply integrating with other models.

Level set methods are effective for tracking moving interfaces and topological changes without explicit parameterization. Their accuracy in interface representation is high, but numerical reinitialization and stability issues increase computational cost. Therefore, they are suitable for problems dominated by boundary migration and topological change, but their application to large-scale three-dimensional systems remains computationally demanding.

## 5. Multi-Coupled Physics Approach

During the formation of crystalline materials, the heterogeneous deformation caused by the different crystallographic orientation of each grain must be considered to obtain an accurate simulation of the microstructure evolution. In the past decade, more and more scholars have combined various methods in the experimental process and applied them to the microsimulation of metals. Raabe [58] systematically reviewed the fundamental principles of cellular automata in materials science, with particular emphasis on their application in recrystallization simulation, showing that this method can describe microstructural evolution, grain boundary migration, and texture development through state variables such as grain orientation, dislocation density, driving force, and neighborhood transition rules. The framework combines a finite element model (CPFEM) based on crystal plasticity with probabilistic cellular automata (CA) methods. The CPFEM part inputs the experimentally measured microstructure information and calculates the evolution of local dislocation density, while the CA model predicts the growth of viable nuclei with high dislocation angles, depending on the difference in storage energy between the nucleus and the surrounding matrix.

**Figure 12:**
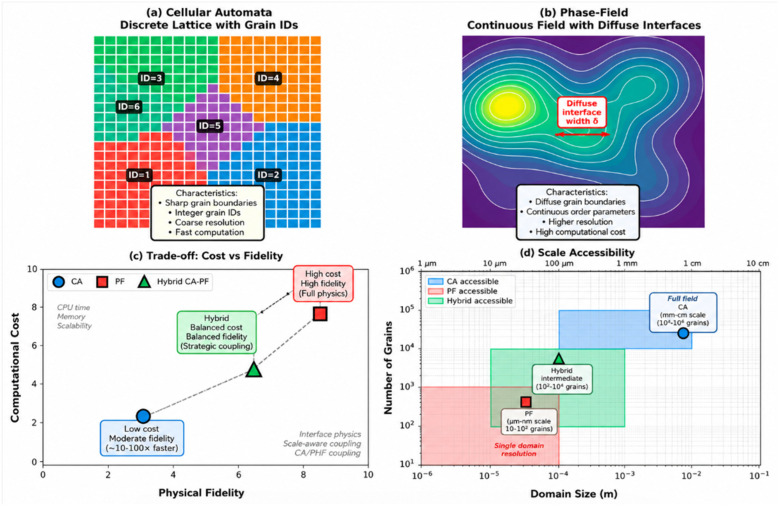
Comparison of cellular automata, phase-field, and hybrid CA–PF methods for microstructure simulation. (**a**) Cellular automata model with discrete lattice-based grain IDs; (**b**) phase-field model with continuous field variables and diffuse interfaces; (**c**) trade-off between computational cost and physical fidelity for CA, PF, and hybrid CA–PF methods; (**d**) accessible simulation scales of different methods in terms of domain size and number of grains [128].

**Figure 13:**
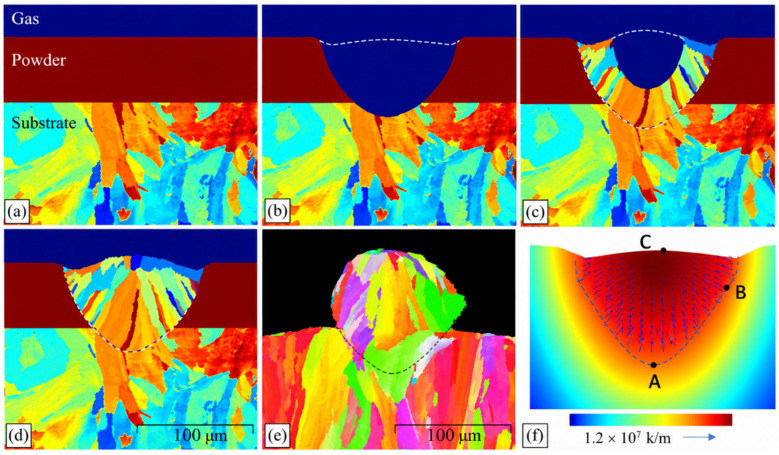
Evolution of melt-pool morphology and microstructure during powder-bed melting. (**a**–**d**) Formation and evolution of the gas–powder–substrate interface and melt-pool profile; (**e**) grain morphology and orientation distribution around the solidified melt pool; (**f**) thermal gradient distribution and heat-flow direction within the melt pool. Region A represents the internal heat-conduction concentration zone, Region B indicates the lateral diffusion transition zone, and Region C corresponds to the high-temperature-gradient region directly affected by the heat source [130].

The CA method and the phase field method can also be shared. Łach, Ł. [128] developed an integrated CA-PF model for predicting dendrical growth in multi-component, multiphase alloys during solidification. The CA model is used to track dendrite growth and associated mass redistribution, while the PF model (reconstructed in a polar coordinate system) is used to calculate the growth dynamics of CA interface units. The model was validated by comparison with analytical models and applied to case studies of casting and laser welding processes, as shown in Figure 12. The model successfully predicts the dendrite morphology and secondary phase formation and distribution of Al-Cu-Mg ternary alloy, which is very close to the experimental results. Shunyu Liu and Yung C. Shin [129] proposed a new 3D cellular automata–phase field (CA-PF) model, which combines 3D cellular automata (CA) and one-dimensional phase field (PF) methods. It is used to predict dendrite formation and evolution efficiently and accurately. The model takes advantage of the high efficiency of the CA model and the high fidelity of the PF model to capture dendrite growth and solute evolution over the entire simulation domain through the CA component, while the PF component is used to calculate the growth dynamics of the solidification front, including growth rate and solute distribution. By coupling the PF component with thermodynamic data, the model can deal with the microstructure evolution of complex multi-component alloys. As shown in Figure 13, a combination of the finite element method and CA method is used in the application diagram [130].

The phase field method and finite element method can also be combined. Ansari Dezfoli et al. [130] adopted the method of combining the phase field method with crystal plasticity theory. The phase field method is used to describe the microstructure evolution during martensitic transformation, including the selection of martensitic variants and the formation of interfaces. The crystal plasticity theory is used to simulate the slip behavior in austenite. This combined method can capture the microstructure evolution within the finite element framework, while taking into account the influence of large deformation and complex stress states. AnupBasak and ValeryI Levitas [131] developed a new multiphase phase field method to simulate martensitic transformation under large strains and interfacial stresses. The researchers used a combination of phase field method and finite element method (FEM). The multiphase field method can naturally deal with the finite width and energy of the interface by introducing multiple order parameters to describe the transition between different phases and variants. The finite element method is used to solve the coupled Ginzburg–Landau equation and the mechanical equilibrium equation, which can handle complex geometric shapes and boundary conditions, while capturing the microstructure evolution under large deformation.

## 6. Molecular Dynamics Method

Molecular dynamics (MD) is an atomistic simulation method that describes the motion and interaction of atoms by solving Newton’s equations of motion. Unlike MC, CA, PF, FEM, and level set methods, MD does not describe microstructure evolution through mesoscopic evolution rules or continuous field variables. Instead, it directly calculates atomic trajectories based on interatomic potentials, allowing microstructural events to emerge from atomic interactions. Therefore, MD is particularly suitable for revealing fundamental mechanisms of grain boundary migration, dislocation nucleation and motion, vacancy diffusion, solute segregation, twinning, and phase transformation [132].

The basic governing equation of MD can be expressed as:

$$m_{i}\;d2r_{i}/dt^{2}=F_{i}=-\partial U/\partial r_{i}$$

where *m_i_* and *r_i_* are the mass and position of atom *i*, respectively, *F_i_* is the force acting on the atom, and *U* is the total potential energy of the atomic system. The accuracy of MD simulations strongly depends on the interatomic potential. Traditional potentials, such as the embedded atom method (EAM) and modified embedded atom method (MEAM), are widely used in metallic systems because of their relatively high computational efficiency. However, their transferability may be limited in complex alloys, non-equilibrium phase transformations, and defect-dominated processes. Recently, machine-learning interatomic potentials have improved the balance between computational efficiency and atomic-scale accuracy, providing new opportunities for more reliable atomistic simulation of complex metallic microstructures.

The main value of MD in microstructure simulation lies in mechanism discovery. Atomic-scale events, such as dislocation emission from grain boundaries, twin boundary migration, local structural rearrangement, defect interaction, and diffusion-controlled interface migration, can be directly observed from atomic trajectories [133,134,135,136]. These mechanisms are difficult to capture experimentally and are often simplified in mesoscale or continuum models. Therefore, MD can provide physical evidence for constructing or validating the assumptions used in CA, PF, level set, and crystal plasticity finite element models. MD also plays an important role in parameter calibration. Parameters such as grain boundary energy, interface mobility, diffusion coefficient, defect formation energy, stacking fault energy, dislocation mobility, and nucleation energy barrier are difficult to obtain directly from experiments, especially under high-temperature, high-strain-rate, or non-equilibrium processing conditions. MD can calculate these parameters under controlled atomic configurations, and the obtained results can be transferred to mesoscale or continuum models. For example, grain boundary energy and mobility obtained from MD can be introduced into phase field or level-set models, while dislocation-related parameters can support crystal plasticity finite element simulations.

However, MD also has obvious limitations. The accessible time scale is usually limited to nanoseconds or microseconds, while many microstructure evolution processes, such as recrystallization and grain growth, occur over much longer time scales. The accessible spatial scale is also much smaller than that of CA, PF, FEM, or level set simulations. In addition, MD simulations often require high strain rates or elevated temperatures to accelerate atomic events, which may deviate from experimental processing conditions. Therefore, MD is not suitable as a standalone method for engineering-scale microstructure prediction. Its most appropriate role is to reveal atomic mechanisms, calibrate key physical parameters, and support multiscale coupling.

## 7. Discussion and Comparison

To move beyond a descriptive comparison, this section establishes a semi-quantitative evaluation framework for different microstructure simulation methods. Four criteria are considered: accuracy, computational cost, scalability, and applicability. Accuracy refers to the ability of a method to reproduce experimentally observed microstructural features, such as grain size, recrystallized fraction, texture evolution, interface morphology, or stress–strain response. Computational cost reflects the relative burden of memory usage, iterative solution, parameter calibration, and simulation time. Scalability describes whether the method can be extended from two-dimensional to three-dimensional simulations, from small domains to large polycrystalline systems, and from single-field to multi-field coupling. Applicability refers to the range of microstructural evolution problems that the method can effectively address, including recrystallization, grain growth, slip, twinning, phase transformation, solidification, and additive manufacturing.

Discrete field model and continuous field model have their own advantages and disadvantages. By discretizing space and state, discrete field models have significant computational efficiency advantages, and can flexibly deal with complex nonlinear relations and local interactions, especially for systems with discrete characteristics. However, its accuracy is limited by the degree of discretization, it is difficult to accurately describe the global characteristics, and its application scope is limited when dealing with continuous change phenomena. In contrast, the continuous field model can accurately describe the phenomenon of continuous change in space, has a wide range of applications and a strong ability to describe global characteristics, and has a solid theoretical foundation. However, its computational complexity is high, the process of model construction and numerical solution is more complex, and it may not be suitable for dealing with systems with discrete characteristics. To clarify the relative advantages and limitations of different simulation approaches, Table 2 provides a semi-quantitative comparison of microstructure simulation methods in terms of prediction accuracy, computational cost, scalability, and applicability. 

The MC model is suitable for describing randomness and local interactions in processes such as recrystallization, grain growth and phase transformation. Cellular automata (CA) can efficiently simulate the spatio-temporal evolution of complex systems by simple local rules, and is suitable for describing the microstructure changes such as dynamic recrystallization, grain growth, slip and twinning. The vertex model can accurately describe the geometry of the grain and the migration behavior of grain boundaries. It is especially suitable for simulating grain growth and phase transition during recrystallization, and can naturally deal with complex topological changes. For the discrete field model, it is difficult to describe the global characteristics precisely due to the degree of discretization, and the statistical error of the calculated results needs to be reduced by a large number of samples and a long time simulation. The CA method can easily set parameters and deal with complex phenomena such as grain nucleation, growth and collision. Therefore, the CA method is relatively widely used in discrete field method.

The phase field method (PF) can naturally deal with the complex microstructure and phase interface, accurately describe the movement of the interface and the morphology of the microstructure, and is suitable for the simulation of metal phase transition, slip and twin. The finite element method (FEM) can accurately handle complex geometries and boundary conditions, and is suitable for simulation from a variety of microscopic angles. The level set model is suitable for the microstructure evolution in processes such as recrystallization and grain growth. Although the continuous field model has the advantage of accurately describing the global characteristics when dealing with complex microstructure evolution problems, its shortcomings of high computational complexity, sensitivity to initial conditions and parameters, and difficulty in dealing with discrete characteristics limit its wide use in some specific applications. In the continuous field method, the phase field method can simulate a variety of metal microscopic changes, which is more complicated than the finite element method and the level set model is too large, so the phase field method is more prominent in the continuous field.

The comparison indicates that no single method is universally optimal for all microstructure evolution problems. MC and CA methods show clear advantages in computational efficiency and large-domain mesoscale simulations, making them suitable for statistical grain growth and recrystallization studies. However, their predictive accuracy is limited by discretization strategy, transition rules, and the difficulty of directly mapping simulation steps to physical time. In contrast, PF and level set methods provide more rigorous descriptions of interface migration and topology evolution. They are therefore more accurate for problems involving phase transformation, dendritic growth, and complex moving boundaries, but their high computational cost restricts their application in large-scale three-dimensional simulations.

FEM and CPFEM are more suitable for problems dominated by mechanical loading, heterogeneous deformation, and thermo-mechanical coupling. Their advantage lies in handling complex geometries, boundary conditions, and constitutive responses, while their limitation is that the evolution of grain boundaries or phase interfaces usually requires coupling with PF, CA, or level set models. ANN-based methods have strong advantages in fast prediction and parameter optimization, especially after training. However, their reliability depends strongly on the quantity and representativeness of training data. Therefore, ANN should not be regarded as a replacement for physics-based models, but rather as an acceleration or surrogate modeling tool.

From the perspective of practical method selection, CA or MC is preferred when the main objective is to simulate grain growth or recrystallization over a large representative volume with acceptable accuracy and low computational cost. PF or level set should be selected when interface morphology, phase boundary migration, or dendritic evolution is the main concern. FEM or CPFEM is more appropriate when stress, strain, and deformation heterogeneity dominate the microstructure evolution. Hybrid methods, such as CA-PF, FEM-PF, and CPFEM-CA, provide the highest physical completeness, but they also introduce higher parameter uncertainty and computational burden. Therefore, the choice of method should be determined by the dominant physical mechanism, required accuracy, available computational resources, and target length scale. To provide practical guidance for method selection, Table 3 summarizes the recommended simulation methods for typical microstructure evolution tasks according to the dominant physical mechanism, simulation scale, and computational requirement.

## 8. Challenges and Future Opportunities

### 8.1. Unresolved Issues

Although the metal microsimulation modeling technology has made remarkable progress in the field of materials science and engineering, there are still some unsolved problems that need to be further studied and explored. When dealing with complex material systems, current microsimulation methods are often difficult to accurately capture their microscopic behaviors and interactions, resulting in insufficient accuracy of model prediction [137]. The implementation of multi-scale simulation is still a challenge, and how to effectively couple atomic-level, micro and macro models to more fully describe the behavior of materials is an urgent problem [138]. Although machine learning shows potential in improving simulation efficiency and accuracy, its application in materials science is still in its infancy, and how to effectively combine machine learning algorithms with physical models still needs further research [138,139]. There are also problems in the open sharing and standardization of data. How to establish a unified data platform to promote the transparency and repeatability of research is the key to promoting the development of the field [140]. In response to these unsolved problems, future research needs to strengthen interdisciplinary cooperation and promote the development of new algorithms and experimental techniques to further enhance the capability and application range of metal microsimulation modeling.

### 8.2. Future Outlook

Metal microstructure evolution modeling technology is facing major challenges of multi-scale, multi-physics coupling and a complex evolution mechanism. Future research and development should focus on the following directions:

(1) The optimization of the cross-scale data transfer algorithm will help to solve the problem of the accuracy loss of parameter mapping on a continuous–discrete interface. The deep integration of physical-driven and data-driven models, such as the combination of the phase field method and a graph neural network to achieve the intelligent prediction of grain boundary migration paths, and the efficient adaptation of heterogeneous computing platforms, will provide new technical support for real-time simulation on the scale of 100 million grids.
(2) Future research directions may include combining metal microsimulation methods with machine learning and extending its application to other related fields. For example, in the machining or cutting process of mechanical parts, the microsimulation method of metal can be used to study its microstructure changes, so as to optimize the processing process and improve the material properties.

## Figures and Tables

**Table 1:** Application history of MC in microscopic simulation.

Year	Proposer	Performance
1973	Hunt [31]	An instantaneous nucleation model is proposed, and it is believed that the nucleus density will reach the maximum value instantaneously when the nucleation temperature is reached.
1986	Oldfield [32]	In simulating the eutectic growth of gray cast iron, a continuous nucleation model was proposed for the first time, which considered that the nucleation is continuously changing, and the number of crystal nuclei maintains a continuous functional relationship with the degree of supercooling.
1999	JL Desbiolles [33]	A quasi-continuous nucleation model is proposed, which is considered to be a continuous gradual process rather than an instantaneous burst process, and the nucleation rate varies with the degree of supercooling in a probability density distribution.
2013	Sieradzki, L [34]	This paper reviews the progress of the microscopic simulation of the solidification microstructure of alloys, and discusses the application of various simulation methods, including the MC method, in the simulation of the solidification microstructure of alloys.
2017	Rodgers T M, Madison J D [35]	The MC method was used to simulate the microstructure evolution of metal additive manufacturing, which provided theoretical support for the optimization of additive manufacturing process.

**Table 2:** Semi-quantitative comparison of microstructure simulation methods.

Method	Accuracy	Computational Cost	Scalability	Applicability	Main Advantages	Main Limitations
Monte Carlo, MC	3.5/5	4/5	3.5/5	3.5/5	Suitable for grain growth, recrystallization and topological evolution with high computational efficiency.	Difficult physical time calibration and weak description capability of local field variables.
Cellular Automata, CA	3.5/5	4.5/5	4/5	4/5	With simple rules, it is applicable to large-scale evolution such as solidification, recrystallization and grain growth.	Simulation results rely heavily on transformation rules, mesh size and neighborhood definition.
Vertex model	4/5	3/5	2.5/5	3/5	It can accurately describe grain boundary geometry, grain topology and interface migration.	Complicated three-dimensional expansion, with high sensitivity to topological reconstruction and parameter settings.
ANN	3.5–4.5/5	5/5	4.5/5	3.5/5	It features fast prediction speed and is suitable for complex nonlinear relationships and parameter optimization.	Dependent on high-quality data, insufficient physical interpretability and limited extrapolation capability.
Phase Field, PF	4.5/5	2/5	3/5	4.5/5	No explicit interface tracking is required, making it suitable for phase transformation, dendrite growth, recrystallization and interface evolution.	High computational cost for 3D simulation and difficult parameter calibration.
FEM/CPFEM	4/5	2.5/5	4/5	4/5	Applicable to complex geometries, boundary conditions and thermo-mechanical coupling problems.	Inferior to the PF method in describing microscale interface evolution, and strongly affected by mesh and constitutive parameters.
Level Set	4/5	2.5/5	3/5	3.5/5	Suitable for interface tracking, grain boundary migration and topological changes.	Requiring reinitialization, with prominent problems in numerical stability and computational cost.
Hybrid methods, CA-PF/FEM-PF/CPFEM-CA	4.5–5/5	1.5–2.5/5	3–4/5	5/5	It can take into account multi-scale, multi-physics field and complex microstructure evolution.	Complex model construction, numerous parameters and high difficulty in model verification.
Molecular Dynamics, MD	4.5/5	1.5/5	2/5	3.5/5	Reveals atomic-scale mechanisms; provides parameters for PF, CA, FEM, and Level Set models; suitable for defects, grain boundaries, diffusion, and dislocation processes	Limited time and length scales; highly dependent on interatomic potentials; difficult to directly predict engineering-scale microstructure evolution

Note: The score ranges from 1 to 5. For accuracy, scalability and applicability, a higher score indicates better performance. For computational cost, a higher score indicates lower computational burden and higher efficiency. The scores are semi-quantitative and are assigned based on the reported validation accuracy, computational complexity, dimensional extensibility, and practical application range in the reviewed literature.

**Table 3:** Recommended method selection for typical microstructure simulation tasks.

Research Objective	Preferred Method	Alternative Method	Methods ot Recommended as Standalone Approaches
Statistical analysis of grain growth	MC/CA	PF/Vertex model	ANN alone
Dynamic recrystallization	CA/MC/CPFEM-CA	PF/FEM-CA	FEM alone
Static recrystallization	MC/CA/Vertex model	PF/Level Set	ANN alone
Phase transformation and dendritic growth	PF	CA-PF/Level Set	MC alone
Thermo-mechanical coupled deformation	FEM/CPFEM	FEM-PF/CPFEM-CA	CA alone
Solidification microstructure in additive manufacturing	CA-PF/PF	FEM-PF/MC	ANN alone
Rapid property prediction and process optimization	ANN/surrogate model	ANN-PF/ANN-FEM	Unconstrained ANN
Atomic-scale mechanism discovery	MD	DFT/MLIP-MD	FEM or CA alone
Parameter calibration for mesoscale models	MD/DFT	MLIP-MD/experiment-informed inverse modeling	Empirical CA or PF without calibration

Note: This selection map indicates that the practical value of each method depends not only on its theoretical completeness, but also on whether its assumptions are consistent with the dominant physical mechanism, target length scale, and available computational resources of the problem being studied.

## Data Availability

No new data were created or analyzed in this study. Data sharing is not applicable to this article.
